# 2212. Cessation of fluoroquinolone prophylaxis in inpatients with haematological malignancy – a retrospective cohort analysis

**DOI:** 10.1093/ofid/ofad500.1834

**Published:** 2023-11-27

**Authors:** Anjaneya Bapat, Lydia N Drumright, Louise Caldwell, Fergus Jimenez-England, Jonathan Lambourne, Samir Agrawal

**Affiliations:** Barts Health NHS Trust, London, England, United Kingdom; University of Washington, Seattle, Washington; Barts Health NHS Trust, London, England, United Kingdom; Barts Health NHS Trust, London, England, United Kingdom; Barts Health NHS Trust, London, England, United Kingdom; Blizard Institute, Queen Mary University of London, London, England, United Kingdom

## Abstract

**Background:**

Fluoroquinolone prophylaxis remains standard-of-care for inpatients with hematological malignancy expected to have prolonged neutropenia. However, there are increasing concerns about drug toxicities, the negative impact on the microbiome, antimicrobial resistance and mortality.

**Methods:**

In our centre, ciprofloxacin prophylaxis was used for patients with acute leukaemia, high-risk myelodysplastic syndrome and those receiving a hematopoietic stem cell transplant until September 2019 (period T1). Thereafter, ciprofloxacin prophylaxis was stopped (period T2). This retrospective cohort analysis analysed 2391 admissions among 1089 patients between April 2017 and January 2022 for outcomes including mortality, Gram-negative bloodstream infection (GNBSI) and admission to the intensive care unit (ICU).

**Results:**

Patients in T1 and T2 were well-balanced for age, gender, underlying diagnosis and treatments (Table 1). The cessation of fluoroquinolone prophylaxis was associated with a significant reduction in mortality (16.7% vs 11.0%, p=0.008), increase in GNBSI (8.5% to 12.4%, p=0.038), but no difference in ICU admissions (8.2 vs 8.3%, p=0.945). Patients in T2 who developed GNBSI had significantly lower rates of fluoroquinolone resistance (RR=0.32; 95% CI: 0.18 -0.56) and extended spectrum beta-lactamase production (RR 0.23; 95% CI: 0.09-0.55). In time updated Cox proportional hazard models, patients in T2 were significantly less likely to die than those in T1 (RR=0.46, 95% CI: 0.37-0.58) in fully adjusted models (Figure 1). The survival benefit seen in T2 patients was seen in both patients with, and without, GNBSI.

Table 1

**Figure:**
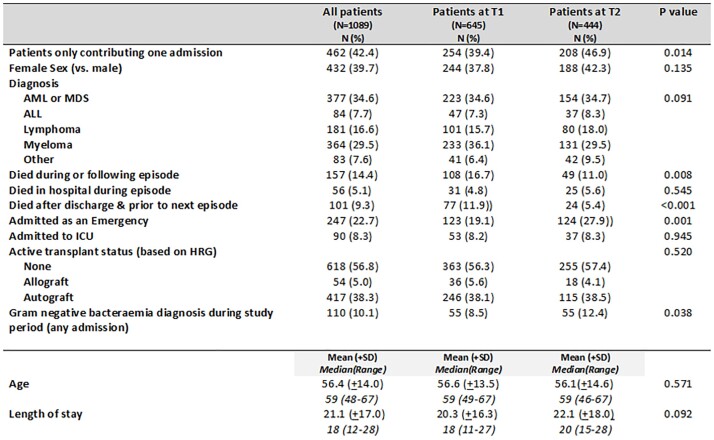

Participant characteristics at first included visit for all ciprofloxacin prophylaxis eligible haemato-oncology inpatients by time period, fluoroquinolone prophylaxis (T1) given and no prophylaxis (T2) given

Figure 1

**Figure:**
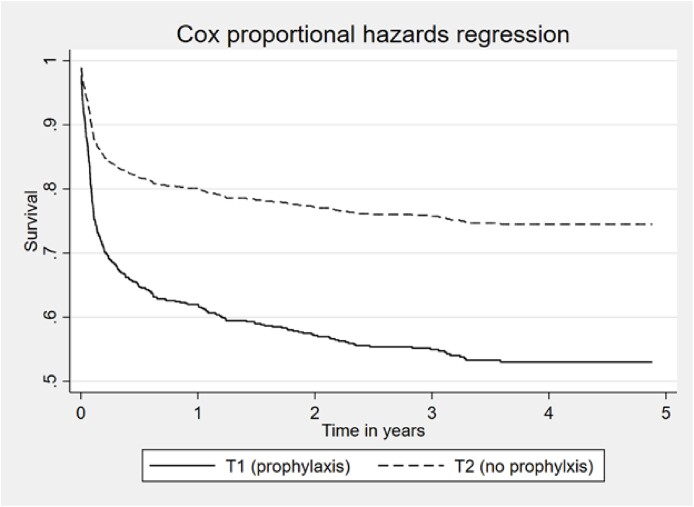

Survival over study time based on contributions to time period 1 with routine ciprofloxacin prophylaxis (T1) and time period 2 with no fluoroquinolone prophylaxis given (T2) among inpatients in haemato-oncology who were fluoroquinolone prophylaxis eligible (n=1089). Fully adjusted models are adjusted for age, sex, admitted through emergency, currently receiving hematopoietic stem cell transplant, cycle of treatment, and diagnosis.

**Conclusion:**

The cessation of fluoroquinolone prophylaxis was associated with significantly lower mortality, lower antimicrobial resistance, no change in ICU admission rates but a higher recorded rate of GNBSI. It is possible that the widespread use of fluoroquinolone prophylaxis may have been causing harms in this patient population.

**Disclosures:**

**Anjaneya Bapat, FRCPath**, Gilead: Honoraria **Jonathan Lambourne, MB BS, BA, MSc, DTM&H FRCP, FRCPath, PhD**, Mundipharma: Advisor/Consultant|Mundipharma: Advisor/Consultant **Samir Agrawal, MD PhD**, Pfizer: Grant/Research Support|Pfizer: Honoraria|Shionogi: Honoraria

